# Mutagenic Effect of ^60^Co γ-Irradiation on *Rosa multiflora* ‘Libellula’ and the Mechanism Underlying the Associated Leaf Changes

**DOI:** 10.3390/plants11111438

**Published:** 2022-05-28

**Authors:** Meng Xia, Qingyu Xu, Ying Liu, Feng Ming

**Affiliations:** 1 Development Centre of Plant Germplasm Resources, College of Life Sciences, Shanghai Normal University, Shanghai 200234, China; summer0501xm@outlook.com (M.X.); 18254932630@163.com (Q.X.); 684724@student.inholland.nl (Y.L.); 2 Shanghai Key Laboratory of Plant Molecular Sciences, College of Life Sciences, Shanghai Normal University, Shanghai 200234, China

**Keywords:** *Rosa chinensis*, ^60^Co-γ-ray irradiation mutagenesis, transcriptome analysis, leaf development

## Abstract

Gamma (γ)-irradiation can induce changes in plant morphology, cellular physiological activities, and genetic material. To date, there has been limited research on the molecular basis of leaf morphological abnormalities and physiological changes in irradiated rose plants. In this study, *Rosa multiflora* ‘Libellula’ plants were treated with ^60^Co γ-rays. The irradiation resulted in the distortion of blade morphology. Additionally, the leaf chlorophyll content decreased, whereas the accumulation of reactive oxygen species increased. The differentially expressed genes between the control and 2–3 plants irradiated with 50 Gy were analyzed by RNA-seq technology, which revealed genes related to chlorophyll metabolism were differentially expressed. The expression levels of genes related to the regulation of antioxidant enzyme synthesis were downregulated. An RNA-seq analysis also identified the differentially expressed regulatory genes involved in leaf morphology development. Four genes (*RcYABBY1*, *RcARF18*, *RcARF9*, and *RcWOX8*) were selected, and their expression patterns in different leaf development stages and in various plant organs were analyzed. Furthermore, virus-induced gene silencing technology was used to verify that *RcYABBY1* is involved in the morphogenesis of *R. multiflora* ‘Libellula’ leaves. The results of this study are useful for clarifying the molecular, physiological, and morphological changes in irradiated rose plants.

## 1. Introduction

*Rosa chinensis*, which belongs to the family Rosaceae, is one of the 10 most famous flowers in China and one of the 4 most popular cut-flower species worldwide. It is an economically valuable ornamental flower species in the cut-flower industry, but it is also used for garden landscaping and the production of oil [1]. Irradiation mutagenesis breeding is now widely used in horticultural crop breeding because of its high variation frequency, large variation spectrum, rapid progeny stability and short breeding life [2]. Cobalt-60 (^60^Co) γ-ray is one of the commonly used irradiation rays. Specifically, γ-irradiation can alter plant morphological characteristics while also inhibiting plant growth and reproductive ability [3]. The damaging effect of gamma rays comes from the direct deposition of ionizing energy and the indirect effect of ionization-induced radiolysis of water on reactive oxygen species (e.g., H_2_O_2_, O_2_, O_2_^−^) produced by macromolecular structures such as cell walls, membranes, and DNA [4,5]. Thus, the γ-irradiation of plants can induce changes to cell physiological activities, genetic material, biochemical reactions, and tissue morphology. Specific changes include the translocation or deletion of DNA fragments [6], the swelling of chloroplast thylakoid membranes [7], decreased plant photosynthetic activities, and the accumulation of phenolic compounds [8]. Among these, chlorophyll content is an important physiological indicator of the degree of physiological damage caused by irradiation in irradiation mutagenesis breeding [9]. It was shown that the concentration of chlorophyll a, chlorophyll b, and total chlorophyll decreased significantly with increasing irradiation dose in γ-ray irradiated soybean breeding [10].

The leaf is an important photosynthetic organ of plants. The leaf morphological structure is closely related to crop yield, but it is also important for plant stress responses [11]. The mechanisms underlying the establishment of the leaf primordium and leaf polarity as well as leaf size are the main determinants of the development and final shape of plant leaves [12]. There are two conserved regulatory mechanisms mediating the initial development of leaf primordia. The first relies on the antagonistic relationship between *KNOTTED1-like Homeobox (KNOXI)* and *ASYMMETRIC LEAVES1 (AS1)* [13]/*ROUGH SHEATH2 (RS2)* [14]/*PHANTASTICA (ARP)* MYB domain transcription factor genes [15]. The second requires *PIN-FORMED1 (Pin1)*, which encodes a protein that transports auxin to the leaf primordium initiation site to ensure leaf development is initiated normally [16,17]. Furthermore, *KNOX* transcription factor genes are important for maintaining the shoot apical meristem (SAM) during development [18].

Adjacent-distal development is important for leaf polarity differentiation. A series of genes encoding functionally redundant HD-ZIPIII transcription factors determines the establishment of leaf adaxial polarity. These genes include *REVOLUTA (REV)* [19], *PHABULOSA (PHB)*, and *PHAVOLUTA (PHV)* [20]. The establishment of leaf abaxial polarity requires the expression of *KANADI (Kan)* genes [21] and genes encoding various auxin-responsive transcription factors [e.g., *AUXIN RESPONSE FACTOR (ARF)*] [22,23]. The YABBY (YAB) transcription factors also help establish leaf abaxial polarity and regulate leaf edge morphological development [24]. In Arabidopsis thaliana, *YAB1*, *YAB2*, *YAB3*, and *YAB5* are expressed on the abaxial side and at the margins of leaves [25]. The Class II *CINCINNATA-like TCP* genes play an important role in determining leaf size. These genes encode a class of plant-specific transcription factors that regulate the transition from cell proliferation to cell differentiation [26,27]. Moreover, *WUSCHEL-related homeobox (WOX)* family genes, which encode transcription factors unique to plants, are crucial for maintaining the stability of stem cell populations in the shoot apex and for regulating floral organ development [28,29].

Leaf morphology is one of the most diverse plant features. The regulation of leaf morphology is directly related to the utilization of light, which in turn affects the quality and yield of horticultural crops. Plant responses to stress are often associated with changes to leaf morphology (e.g., curling, serrated nicks at the margins, decreased width and overall size, and development of a lanceolate phenotype). The molecular mechanism regulating the deformation of the leaves of rose plants exposed to stress, including irradiation, remains to be characterized. Therefore, to better select and breed excellent *Rosa* varieties, investigating the mutation mechanism after irradiation treatment can provide theoretical support and effective guidance for breeding work.

*Rosa multiflora* ‘Libellula’, which is a rose species that produces blue--purple flowers, was first cultivated by Japanese breeder Kiyomi Imai in 2007. Its popularity is related to the desirable shape and strong fragrance of its flowers [30]. In this study, *Rosa multiflora* ‘Libellula’ plants were treated with 40 and 50 Gy ^60^Co γ-rays, which resulted in nicked, curled, or asymmetric leaves as well as a decrease in the number of compound leaves. The differentially expressed genes (DEGs) in the irradiated and control plants were analyzed by RNA-seq technology. Additionally, the DEGs related to the establishment of the leaf primordium and leaf polarity as well as leaf size were identified. The expression patterns and functions of the related genes were verified. The objective of this study was to provide a technical reference for the γ-irradiation-based mutagenesis of rose varieties and the development of new germplasm, with implications for future research on rose leaf morphogenesis and artificial breeding.

## 2. Results

### 2.1. Morphological Deformation of Rosa multiflora ‘Libellula’ Leaves Treated with ^60^Co γ-rays

**Figure 1:**
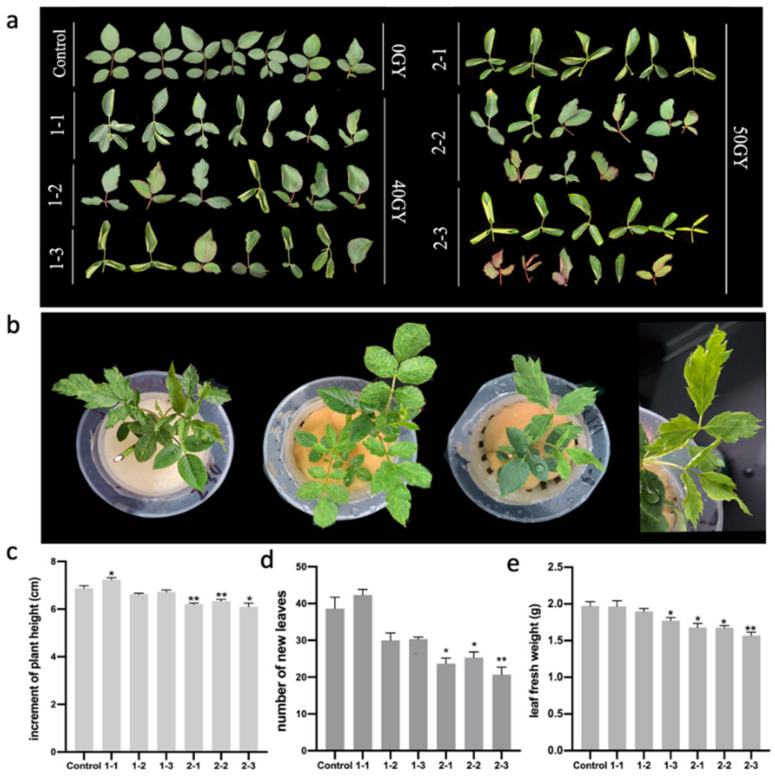
Effects of different doses of ^60^Co γ-rays on the *Rosa multiflora* ‘Libellula’ leaf phenotype and growth record of the plants: (**a**) differences in the leaf phenotype between the control leaves and the leaves irradiated with 40 and 50 Gy; (**b**) deformed leaves of hydroponic *R. multiflora* ‘Libellula’ seedlings irradiated with 50 Gy; (**c**) Increment of plant height on the 30th day after irradiation; (**d**) the number of new leaves of the plants on the 30th day after irradiation; (**e**) the leaf fresh weight of the plants on the 30th day after irradiation. Asterisks indicate significant differences among treatments (* *p* < 0.05; ** *p* < 0.01). The vertical bars indicate the standard deviations of the means of three tests.

In this study, *R. multiflora* ‘Libellula’ biennial cuttings, which were treated with 40 and 50 Gy ^60^Co γ-rays, were used as experimental materials. The irradiated plants exhibited stable growth and produced new leaves after 60 days of incubation (Table 1, Figure S2). The main leaf-related changes caused by irradiation were as follows: leaf nicks (mesophyll development was restricted, resulting in shallow, semi-lobed, deep-lobed, and sharp-cleft gaps), decrease in the number of compound leaves, leaf curling (shrunken and rolled inward), leaf expansion (significant increase in the leaf area), development of bicuspid leaves (two leaf tips), and asymmetric leaves (half of the leaves developed normally, and half were missing or deformed) (Figure 1a,b). These changes were most obvious for the 2-3 leaves following the 50 Gy irradiation treatment. When *R. multiflora* ‘Libellula’ hydroponic seedlings were irradiated with 50 Gy ^60^Co γ-rays, the new leaves of the irradiated plants were severely notched, asymmetric, and wrinkled.

The growth and development of the plants were observed and recorded after irradiation treatment. The plant height was measured on the 30th day after irradiation, and we found that the 1-1 had the highest increment, with an increase of about 5.19% compared to the control, while the number of its new leaves sprouted was 43, which was slightly increased compared to the control plants (Figure 1c,d). The growth of plants in the 50Gy irradiation treatment group was significantly inhibited. Specifically, compared to the control, the increment of plant height of the 2-1 and 2-2 significantly decreased by about 9.84% and 7.95%, respectively (Figure 1c). The number of the 2-3 new leaves was 21, which decreased by about 46.15% compared to the control (Figure 1d). Fresh leaves from the upper middle part of the plant that were fully expanded were punched into discs of uniform size using a hole punch. Afterwards, 20 leaflet discs were weighed (Figure 1e). We found that the fresh weight of the leaves was reduced after irradiation treatment, with the most significant decrease in the 2-3, which was about 20.41% compared to the control group.

### 2.2. Irradiation of Plants with ^60^Co γ-rays Increased the Accumulation of Active Oxygen and Decreased the Chlorophyll Content

**Figure 2:**
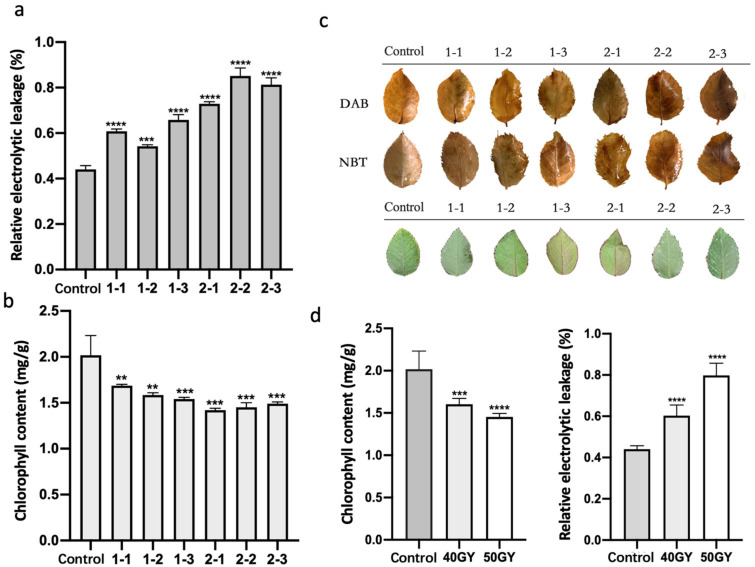
Physiological and biochemical indices of leaves from *Rosa multiflora* ‘Libellula’ plants irradiated with ^60^Co γ-rays: (**a**) relative electrolytic leakage in leaves; (**b**) leaf chlorophyll contents; (**c**) DAB and NBT staining results and changes in chlorophyll accumulation; (**d**) total chlorophyll contents and relative electrolytic leakage after the 40 and 50 Gy irradiation treatments. Asterisks indicate significant differences among treatments (** *p* < 0.01; *** *p* < 0.001; **** *p* < 0.0001). The vertical bars indicate the standard deviations of the means of three tests.

An exposure to radioactivity can directly affect tissues or cells, resulting in toxic effects, but it can also affect biomolecules in cells, especially water molecules, leading to the production of reactive oxygen species (ROS), including superoxide anion (O_2_^•–^) [5], hydroxyl radical (•OH) [31], singlet oxygen (^1^O_2_), and hydrogen peroxide (H_2_O_2_) [32]. The oxidative stress due to the excessive accumulation of ROS may lead to cell death and even plant death. To examine the accumulation of ROS in rose leaves treated with ^60^Co γ-rays, the leaves were stained with diaminobenzidine (DAB) and nitrotetrazolium blue (NBT) staining after irradiation treatment (Figure 2c). The staining results revealed that the ^60^Co γ-irradiation increased the accumulation of ROS in leaves in a dose-dependent manner. An analysis of the relative electrolytic leakage of the leaves of irradiated plants (Figure 2a) indicated the leakage of leaf cell electrolytes increased significantly after the ^60^Co γ-irradiation, indicative of a seriously damaged membrane system. Additionally, the relative electrolytic leakage of leaves irradiated with 50 Gy differed significantly from the relative electrolytic leakage of the control leaves (Figure 2d).

The chlorophyll content is an important indicator of plant photosynthetic activities involving the absorption, transmission, and conversion of light energy. High-dose irradiation (100–500 Gy) leads to growth inhibition, chlorophyll degradation, morphological distortion, and plant death [33]. Unlike the leaves in the control group, the leaves of rose plants treated with ^60^Co γ-rays were leathery and tarnished (Figure 2c). The 60-day irradiation treatment resulted in a significant decrease in the leaf chlorophyll content (Figure 2b). The decrease was greater for the 50 Gy irradiation than for the 40 Gy irradiation (Figure 2d).

### 2.3. RNA-Seq Analysis of Differentially Expressed Genes in Irradiated Rose Leaves

**Figure 3:**
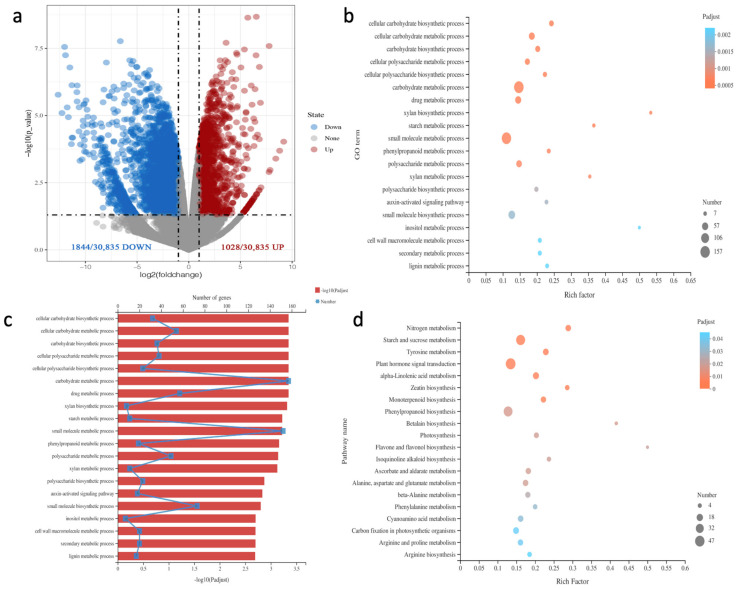
Differentially expressed genes (DEGs) are identified by RNA-seq and their functional annotation: (**a**) volcano plot of DEGs. The significantly up-regulated and down-regulated genes are in red and blue, respectively (*p* < 0.05); (**b**) gene ontology (GO)-based functional annotation of 2872 DEGs; 20 representative pathways are listed; (**c**) tthe number of genes in each GO pathway and results of the *p*-value analysis; (**d**) enriched KEGG pathways among 2872 DEGs; 20 representative pathways are listed.

To elucidate the mechanisms underlying the changes in the leaf morphology, physiological activities, and biochemical reactions of plants treated with ^60^Co γ-rays, we selected the 2-3 and control leaves with significantly different characteristics for RNA-seq analysis. Principal components analysis (PCA) was performed to show the similarity of replicates (Figure S3). A total of 2872 DEGs between the 2-3 and control leaves were detected, of which 1028 were up-regulated and 1844 were down-regulated (Figure 3a). The gene ontology (GO) analysis of the DEGs indicated they were mainly involved in the following: cellular carbohydrate biosynthetic process, cellular carbohydrate metabolic process, carbohydrate biosynthetic process, cellular polysaccharide metabolic process, cellular polysaccharide biosynthetic process, carbohydrate metabolic process, drug metabolic process, xylan biosynthetic process, starch metabolic process, small molecule metabolic process, and phenylpropanoid metabolic process (Figure 3b,c). The main enriched KEGG pathways among the DEGs were as follows: nitrogen metabolism, starch and sucrose metabolism, tyrosine metabolism, plant hormone signal transduction, alpha-linolenic acid metabolism, zeatin biosynthesis, monoterpenoid biosynthesis, phenylpropanoid biosynthesis, betalain biosynthesis, and photosynthesis (Figure 3d).

### 2.4. Antioxidant Functions in Irradiated Plants Were Weakened, and the Genes Related to Chlorophyll Synthesis and Metabolism Were Differentially Expressed

**Figure 4:**
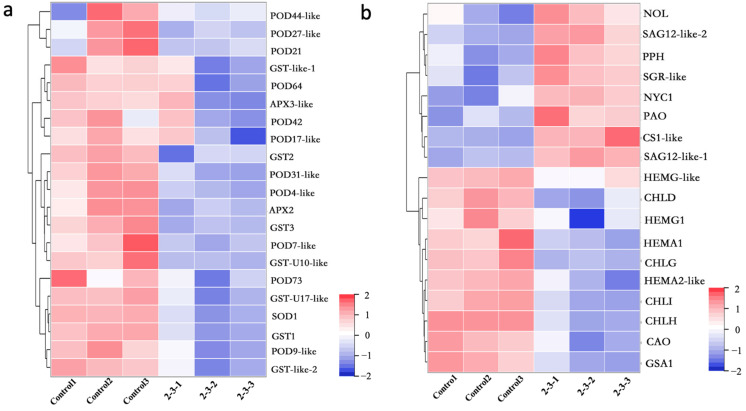
Differentially expressed genes (DEGs) related to the reactive oxygen species metabolic pathway and the chlorophyll metabolic pathway. The gene-related information for the heat map analysis is included in Tables S4 and S5: (**a**) heat map analysis of the DEGs (between the 2-3 and control plants) that encode antioxidant enzymes; (**b**) heat map analysis of the DEGs (between the 2-3 and control plants) that encode chlorophyll synthase and chlorophyll-metabolizing enzymes.

To clarify why ROS accumulated in the ^60^Co γ-irradiated leaves, the identified DEGs related to the regulation of antioxidant enzyme synthesis were analyzed. Compared with the control leaves, the expression levels of POD synthesis genes (e.g., *POD44-like*, *POD27-like*, *POD21*, *POD64*, *POD42*, *POD17-like*, *POD73*, and *POD4-like*) were significantly down-regulated in the 2-3 leaves. Similarly, the expression levels of SOD synthesis genes (e.g., *SOD1*), APX synthesis genes (e.g., *APX3-like* and *APX2*), and glutathione S-transferase synthesis genes (e.g., *GST-like-1*, *GST2*, *GST3*, *GST1,* and *GST-like-2*) were also significantly down-regulated (Figure 4a). The down-regulated expression of these genes induced by the ^60^Co γ-irradiation treatment may have contributed to the observed accumulation of ROS in rose leaves.

Because the irradiated plants had leathery leaves with decreased chlorophyll contents, the expression of the DEGs encoding chlorophyll-degrading enzymes was analyzed. Compared with the corresponding expression in the control leaves, *NOL*, *PPH*, *SGR-like*, *NYC1*, *PAO*, and *CS1-like* were more highly expressed in the 2-3 leaves (Figure 4b), indicative of an increase in the abundance of chlorophyll-degrading enzymes in the leaves of the irradiated plants. Furthermore, *Senescence Associated Gene (SAG)* is a specific marker gene for leaf senescence. We observed that the *SAG12* expression level was much higher in the 2-3 leaves than in the control leaves. To determine whether the irradiation treatment inhibited plant chlorophyll synthesis, the expression of the genes encoding key enzymes catalyzing chlorophyll synthesis was examined (Figure 4b). The expression levels of *HEMA1* and *HEMA2-like* (encoding glutamyl-tRNA reductase), *HEMG-like* (encoding glutamate-1-semialdehyde 2,1-aminomutase), *HEMG* (encoding protoporphyrinogen oxidase), *CHLD*, *CHLI*, *CHLH* (encoding magnesium chelatase H subunit), *CHLG* (encoding chlorophyll synthase), and *CAO* (encoding chlorophyllide a oxygenase) were significantly lower in the 2-3 leaves than in the control leaves. Accordingly, we speculated that the decrease in the chlorophyll content of rose plants after the ^60^Co γ-irradiation was the result of inhibited chlorophyll synthesis and enhanced chlorophyll degradation.

### 2.5. Differential Expression of Genes Related to Leaf Development and Morphogenesis in Irradiated Plants

**Figure 5:**
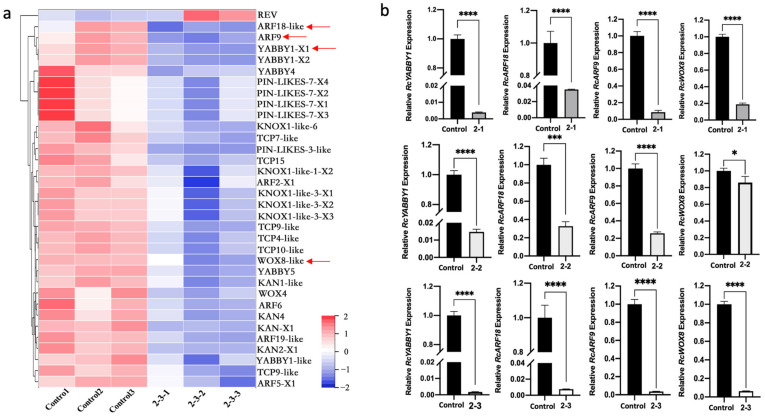
Screening and expression analyses of differentially expressed genes (DEGs) related to leaf development and morphogenesis: (**a**) heat map analysis of DEGs (between 2-3 and control) associated with leaf development and morphogenesis; genes used for the qPCR validation are marked with arrows; (**b**) qPCR analysis of DEGs. Asterisks indicate significant differences among treatments (* *p* < 0.05; *** *p* < 0.001; **** *p* < 0.0001). The vertical bars indicate the standard deviations of the means of three tests.

To explore how exposure to radiation leads to leaf morphological abnormalities, the expression of DEGs identified as key regulatory genes related to leaf size and the establishment of leaf primordia and leaf polarity was analyzed (Figure 5a). The expression levels of *KNOX1-like-6* and *KNOX1-like-3*, which contribute to the formation of leaf primordia, were significantly down-regulated in the 2-3 plants. The expression levels of *PIN-LIKES-7* and *PIN-LIKES-3*, which encode auxin transporters, were also significantly lower in the irradiated plants than in the control plants, implying auxin transport was inhibited in the irradiated plants, which is detrimental to the initiation and development of leaf primordia. The DEGs included many regulatory genes related to the establishment of leaf polarity, especially adaxial polarity. For example, the *REV* gene, which encodes an HD-ZIP III transcription factor that establishes paraxial polarity, was expressed at significantly higher levels in 2-3 plants than in control plants. In contrast, the KANADI-encoding genes (*KAN1-like*, *KAN2*, and *KAN4*) and other genes associated with leaf abaxial polarity had significantly down-regulated expression levels in the 2-3 plants. Similarly, *YABBY1*, *YABBY4*, and *YABBY5*, which also help establish leaf abaxial polarity, were expressed at lower levels in the 2-3 plants than in the control plants. Additionally, the expression levels of auxin-responsive transcription factor genes (*ARF18-like*, *ARF9*, *ARF19-like*, *ARF6, ARF5*, and *ARF2*) as well as *WOX8-like* and *WOX4*, which are important for establishing leaf polarity and leaf growth, were down-regulated in 2-3 plants, which may have contributed to the observed abnormalities in leaf polarity and leaf morphogenesis. In terms of the genes associated with leaf size, the expression of *TCP7-like, TCP9-like, TCP4-like, TCP10-like,* and *TCP15* was down-regulated in the 2-3 plants.

The DEGs *KNOX1-like, ARF18-like, ARF9, YABBY1*, and *WOX8* were selected for a qPCR analysis, which indicated the expression of these genes was down-regulated in the 2-3 leaves (Figure 5b and Figure S4). To assess whether the abnormal leaf phenotypes caused by the ^60^Co γ-irradiation were due to the down-regulated expression of these genes, we examined deformed leaves from plants that underwent the 50 Gy irradiation treatment. Compared with the gene expression in control leaves, only *ARF18-like, ARF9, YABBY1,* and *WOX8* had similar significantly down-regulated expression levels in the 2-1, 2-2, and 2-3 leaves (Figure 5b). The qPCR analysis verified the reliability of the RNA-seq data. We speculated that the down-regulated expression of *ARF18-like, ARF9, YABBY1*, and *WOX8* in response to the ^60^Co γ-irradiation treatment may have affected plant leaf morphogenesis, ultimately resulting in deformed leaves.

### 2.6. Identification and Functional Analysis of Candidate Genes for Leaf Development in Rosa multiflora ‘Libellula’

**Figure 6:**
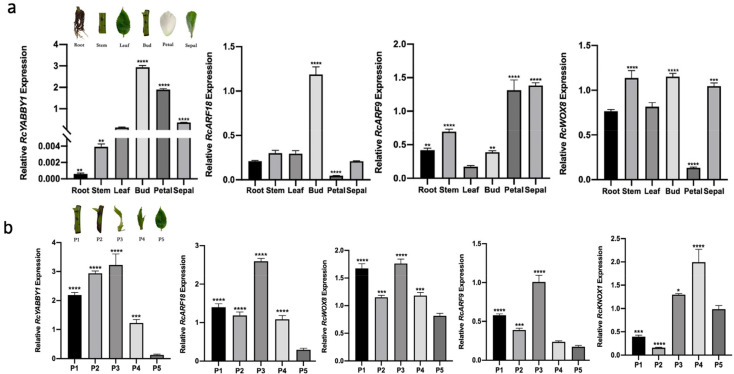
Expression patterns of candidate genes *RcYABBY1, RcARF18, RcARF9,* and *RcWOX8* in *Rosa multiflora* ‘Libellula’: (**a**) expression patterns in different organs of *R. multiflora* ‘Libellula’; (**b**) expression patterns in *R. multiflora* ‘Libellula’ leave at different developmental stages. Asterisks indicate significant differences among treatments (* *p* < 0.05; ** *p* < 0.01; *** *p* < 0.001; **** *p* < 0.0001). The vertical bars indicate the standard deviations of the means of three tests.

Based on the RNA-seq and qPCR data, we speculated that the down-regulated expression of *ARF18-like, ARF9, YABBY1*, and *WOX8* after the ^60^Co γ-irradiation may adversely affect leaf morphogenesis. To further elucidate the functions of these genes, their expression patterns in different *R. multiflora* ‘Libellula’ organs were determined by qPCR (Figure 6a). The *RcYABBY1* expression level was highest in the leaf buds, followed by the petals, sepals, and leaves, whereas the gene was expressed at low levels in the roots and stems. The *RcARF18* expression levels were highest and lowest in the leaf buds and petals, respectively. This gene was similarly expressed in the roots, stems, leaves, and sepals. The expression of *RcARF9* was highest in the petals and sepals, whereas it was lowest in the leaves. In contrast, *RcWOX8* expression was highest in the stems, leaf buds, and sepals, whereas it was lowest in the petals.

The leaf development stage (from leaf buds to mature leaves) of *R. multiflora* ‘Libellula’ was divided into five key periods (P1–P5). A comparison of the expression levels of related genes throughout the leaf development stage (Figure 6b) revealed the *RcYABBY1* expression level was relatively high from P1 to P3, after which it gradually decreased. The expression levels of *RcARF18* and *RcARF9* initially decreased and then increased (P1–P3) and then decreased again (P4 and P5). The *RcWOX8* expression level fluctuated considerably from P1 to P5 (decreased, increased, and then decreased). The *RcYABBY1, RcARF18, RcARF9,* and *RcWOX8* expression levels were higher in P3 than in P5.

**Figure 7:**
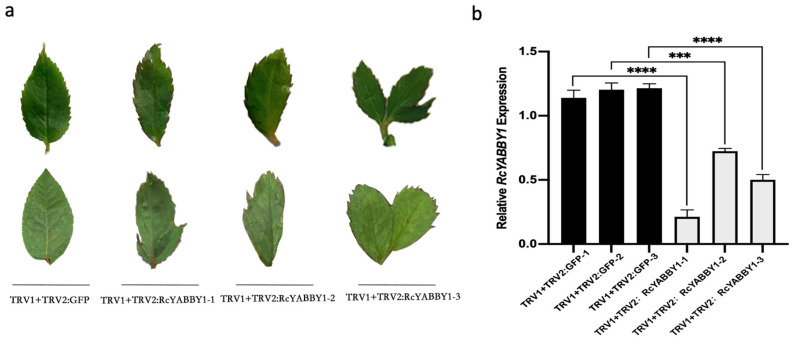
Silencing of *RcYABBY* in *Rosa multiflora* ‘Libellula’: (**a**) leaf phenotypes of *R. multiflora* ‘Libellula’ TRV1 + TRV2:GFP (control) and TRV1 + TRV2:RcYABBY1 (RcYABBY1 silenced); (**b**) qPCR analysis to determine gene silencing efficiency. Asterisks indicate significant differences among treatments (*** *p* < 0.001; **** *p* < 0.0001). The vertical bars indicate the standard deviations of the means of three tests.

Because the *RcYABBY1* expression level had an increasing trend from P1 to P5, especially in the first three leaf development periods, we speculated that *RcYABBY1* may encode a protein with an important function during the initial establishment of leaf morphology. To verify the role of *RcYABBY1* in leaf development and morphogenesis, its expression in *R. multiflora* ‘Libellula’ leaves was silenced via TRV-based virus-induced gene silencing (Figure 7b). The expression of *RcYABBY1* decreased significantly in the TRV1 + TRV2:RcYABBY1 lines. In contrast to TRV1 + GFP, the TRV1 + TRV2:RcYABBY1 lines had serrated leaf margins, asymmetric leaves, and bicuspid leaves (Figure 7a).

## 3. Discussion

Using ^60^Co-γ-rays irradiation to breed roses has the characteristics of a simple experimental method, breaking gene linkage, short breeding years, obtaining mutants in a short time, and selecting new varieties efficiently. Gamma rays can cause various morphological and physiological changes in plants through direct and indirect interactions with organisms, such as abnormal appearance, reduced growth, and reduced reproductive capacity [34]. It also can cause physiological changes such as accumulation of anthocyanins [35], expansion of root cells [36], changes in photosynthesis [37], dysregulation of antioxidant systems, etc. [7,38]. In this study, *R. multiflora ‘Libellula’* biennial cuttings were irradiated with ^60^Co-γ rays, and two irradiation doses of 40 GY and 50 GY were performed. The leaves of the irradiated rose plants were distorted (Figure 1). There were various types of variation, such as leaf nicks, reduced number of compound leaves, leaf curling, and asymmetric leaf development (Table 1). Physiological indexes of leaves of irradiated rose plants were analyzed. DAB and NBT staining results showed that there was more ROS in leaves. Meanwhile, the increase in relative electrolytic leakage of irradiated rose plants showed that their membrane system was seriously damaged. We also measured the chlorophyll content in the leaves and found that the irradiation treatment significantly reduced the chlorophyll content in the leaves (Figure 2).

To cope with the complex external environment, plants have formed a complex ROS balance regulation mechanism during the evolution process. The enzymatic scavenging mechanism of ROS in plants mainly includes superoxide dismutase (SOD), ascorbate peroxidase (APX), glutathione peroxidase (GPX), catalase (CAT), and glutathione S-transferases (GST). Furthermore, peroxidase (POD) can be expressed in the early stages of adversity or senescence, removes H_2_O_2_, and is a member of the cellular reactive oxygen species protection enzyme system [39]. In studies on plant cells, antioxidant enzyme activity is closely related to plant self-metabolism and plant stress [40]. By RNA-seq analysis of the DEGs of the irradiated plants and the control group, we found that the expression levels of genes related to antioxidant enzyme synthesis were significantly reduced in irradiated leaves. POD enzyme synthesis genes such as P*OD44-like, POD27-like, POD21, POD64, POD42, POD17-like*, *POD73, POD4-like,* and other genes were significantly down-regulated. SOD enzyme synthesis genes such as *SOD1*, APX enzyme synthesis genes such as *APX3-like, APX2,* GST enzyme synthesis genes such as *GST-like-1, GST2, GST3, GST1, and GST-like-2* were significantly down-regulated (Figure 4). This may have led to a high accumulation of ROS in plant leaves after irradiation.

Chlorophyll degradation is regulated by enzymatic metabolism in receptors. The degradation of chlorophyll is regulated by enzyme metabolism in plants. Six Chl catabolic enzymes (CCEs) have been identified in *Arabidopsis* and *Rice*, including *NON-YELLOW COLORING1 (NYC1)* and *NYC1-LIKE (NOL)*-encoded chlorophyll b reductase, 7-Hydroxymethyl chlorophyll a reductase (HCAR), pheophytinase (PPH), pheide a oxygenase, (PAO) and red chlorophyll catabolite reductase (RCCR) [41,42]. In addition to the above significant enzyme metabolism genes, STAY-GREEN (SGR) and METHYLESTERASE FAMILY MEMBER 16 (MES16) are also involved in the regulation of chlorophyll degradation in the Arabidopsis PAO pathway [43]. Moreover, chlorophyllase (CS) can catalyze the degradation of chlorophyll a [44]. RNA-seq analysis showed that the expression levels of *NOL, PPH, SGR-like, NYC1, PAO,* and *CS1-like* synthetic genes in the leaves of the irradiated plants were significantly higher than those of the control group. Irradiated leaves may contain more chlorophyll degrading enzymes, which degrade chlorophyll and reduce the chlorophyll content of leaves (Figure 4).

There are 15 steps in chlorophyll biosynthesis from L-glutamyl-tRNA to chlorophyll a and chlorophyll b, involving 15 enzyme-catalyzed reactions, and mutation of any enzyme gene or inhibition of its activity may lead to changes in chlorophyll content, which may affect photosynthetic efficiency and even lead to plant death. The key enzymes in this process are glutamyl-tRNA reductase (GluTR), glutamate-1-semialdehyde 2,1-aminomutase (GSA-AM), protoporphyrinogen oxidase (PPOX), magnesium chelatase H subunit (MgCh), magnesium chelatase I subunit (MgCh), chlorophyll synthase (CHLG), chlorophyllide a oxygenase (CAO), etc. [45]. In the irradiated plants, The expression levels of *HEMA1* and *HEMA2-like* (encoding glutamyl-tRNA reductase), *HEMG-like* (encoding glutamate-1-semialdehyde 2,1-aminomutase), *HEMG* (encoding protoporphyrinogen oxidase), *CHLD, CHLI, CHLH* (encoding magnesium chelatase H subunit), *CHLG* (encoding chlorophyll synthase), and *CAO* (encoding chlorophyllide a oxygenase) were significantly lower than that in control, indicating that chlorophyll synthesis in leaves was inhibited after the plants were irradiated with ^60^Co-γ rays (Figure 4).

Given the leaf aberration phenotype of irradiated plants, RNA-seq analysis showed that in the leaves of irradiated plants, the genes involved in encoding the KNOX protein [14] were significantly down-regulated, and the expression level of genes encoding the Pin1 protein [17] was also considerably lower than that in control. The expression of *REV*, a transcription factor that determines the establishment of blade adaxial polarity [19], was significantly higher in irradiated plants than in control, and the expression levels of Kan gene family genes [46] and YAB gene family related genes [24] involved in the establishment of the abaxial polarity of leaves were significantly downregulated. In addition, the expression levels of ARFs and WOXs genes [22,28] that play an essential role in the establishment of leaf polarity and leaf growth were significantly reduced, which may lead to the loss of leaf polarity and abnormal development in leaf morphogenesis, resulting in aberrant phenotypes in leaves. In terms of leaf size formation, we found that TCP transcription factors of the TCP gene family [26,27] were downregulated in irradiated plants. In the transcriptome, we selected *ARF18-like, ARF9, YABBY1,* and *WOX8*, which are closely related to leaf development and polarity establishment, for qPCR analysis, and their expression levels were the same in plants 2-1, 2-2, and 2-3, a significant downward trend. This indicates that the down-regulated expression of these genes after ^60^Co-γ-ray irradiation may affect plant leaf morphogenesis, which leads to the appearance of aberrant phenotypes and proves the reliability of the RNA-seq data (Figure 5). Afterward, we analyzed the expression of *RcYABBY1, RcARF18, RcARF9,* and *RcWOX8* in different leaf development stages and organs of *R. multiflora* ‘Libellula’ (Figure 6). Finally, the role of RcYABBY1 in the leaf development and morphogenesis of *R. multiflora* ‘Libellula’ was also verified. The silent lines showed the phenotypes of serrated edge deepening, asymmetric leaf growth, and leaf adhesion growth with cracks (Figure 7). This indicates that this gene plays a vital role in the development and morphogenesis of *R. multiflora* ‘Libellula’. It also confirms the down-expression of genes such as *RcYABBY1* after ^60^Co-γ irradiation treatment may eventually lead to morphological aberrations in leaves.

## 4. Materials and Methods

### 4.1. Plant Material Culture Conditions and Irradiation Treatment Methods

*Rosa multiflora* ‘Libellula’ biennial cuttings were used in this study. They were grown in the cultivation greenhouse of the Germplasm Resources Development Center of Shanghai Normal University. The plants with the same growth condition were selected for irradiation treatment. The plants were irradiated at the Beam Energy Irradiation Center of the Shanghai Academy of Agricultural Sciences (Figure S1). We set the 40 and 50 Gy doses for ^60^Co-γ irradiation with a rate of 150 GY∙h^−1^ (three plants per irradiation dose). The plants with an irradiation dose of 40 GY were named 1-1, 1-2, 1-3, and the plants with an irradiation dose of 50 GY were named 2-1, 2-2, and 2-3. The untreated and irradiated plants were cultured and observed in the same condition. After plant growth was stable (30 days), plants were used for the subsequent analysis and RNA-seq of the physiological indexes.

### 4.2. Determination of Physiological Indicators

Determination of chlorophyll content: 0.2 g of the middle and upper fully expanded leaves of each plant were collected, removed from the thick veins, cut into pieces, ground in 80% acetone, and the homogenate was centrifuged at 1000× *g* for 5 min. The absorbance of the supernatant was determined at 663 and 645 nm by a spectrophotometer UV-2550. Chl a, Chl b, and total Chl contents were calculated according to the method of Arnon [47].

Diaminobenzidine (DAB) and nitrotetrazolium blue (NBT) staining: take control plants or leaves of irradiated plants as samples, add DAB or NBT staining solution (both liquids were not over the samples), heat the water bath at 100 °C for 5 min, leave the staining solution for 16 h in a place protected from light, pour out the staining solution, wash the samples with distilled water 2~3 times, add anhydrous ethanol, heat the water bath for 5 min, pour out the ethanol, repeat until decolorization, add 40% volume fraction of glycerol solution, leave the solution in a place protected from light for 2 h, pour out the glycerol solution, and observe [48].

Relative electrolytic leakage determination: the pumping method was used. Four leaves of uniform size were taken as a group with a puncher, and three replicates of each sample were added with 10 mL ddH_2_O and evacuated until the leaves were all submerged in water. Shake the shaker lightly for 4 h and then measure the conductivity meter for C1 before cooking with a conductivity meter. Boiling water bath for 20min, after cooling to room temperature, measure the conductivity C2 after cooking. Relative electrolytic leakage = C1/C2 × 100% [48].

### 4.3. RNA Extraction and Quantitative Real-Time PCR Detection

The roots, stems, leaves, buds, petals, sepals, and leaves of the biennial cuttings of *Rosa multiflora* ‘Libellula’ were harvested to detect leaf development-related gene expressions. Five key stages of leaf development of *Rosa multiflora* ‘Libellula’ (P1–P5) were gathered to detect leaf development-related gene expressions. The newly growing leaves of the control and VIGS rose plants (three to four weeks old) were harvested for silencing effect identification. Total RNA was extracted using a SteadyPure Plant RNA Extraction Kit (Code: AG21019; Accurate Biotechnology Co., Ltd., Hunan, China); cDNA synthesis was performed with the RT reagent kit (Takara) according to the manufacturer’s protocol.

Real-time PCRs were obtained on a Chromo 4™ continuous fluorescence detector with the SYBR RT-PCR Kit (Takara), 20 µL reaction volume: 10 µL of SYBR Green I PCR mix, 0.5 µM forward and reverse primers, 1 µL of cDNA template and supplemented with the appropriate amount of ddH2O to 20 µL. Amplification conditions were: 2 min at 95 °C; 40 cycles of 15 s at 95 °C, 30 s at 58 °C, and 30 s at 72 °C. Fold changes of RNA transcripts were calculated by the 2^−∆∆Ct^ method [49]. We used RcUBC (LOC112195044) in *R.* *Chinensis* plants as the internal reference. The entire experiments were repeated three times, and primers used in the qPCR experiments are listed in Supplementary Table S1 (Table S1).

### 4.4. Functional Verification of Virus-Induced Gene Silencing (VIGS)

*Rosa multiflora* ‘Libellula’ annual cuttings were used in the experiment. The part of the homologous nucleotide sequence of *RcYABBY1* in *R. multiflora* ‘Libellula’ was amplified using primers in Supplementary Table S1 and determined by Tsingke Biotechnology Co., Ltd. (Shanghai, China). Then, the gene fragments were separately linked to the pTRV2 viral vector. The pTRV2 vectors constructed above were introduced into *A. tumefaciens* strain GV3101. Then, TRV1 and *Agrobacterium* carrying the TRV2-derived vector of the target gene were mixed 1:1, and the plants were infected by vacuuming and the bacterial broth without the target gene fragment as the control (TRV1 + TRV2:GFP). The infection solution was placed in a sterilized tissue culture bottle, the plants’ roots were immersed then put them in a vacuum pump to evacuate to −0.098 Mpa, maintained for 5 min, and repeated the above pumping steps three times. After inoculation, the plants were placed in a humidified incubator at 4 °C for 96 h in the dark and then transplanted and cultured at 20–25°C for three to four weeks. The newly growing leaves were harvested for phenotypic identification and silencing effect identification.

### 4.5. RNA-Seq Data Processing, Reassembly, and Annotation

RNA purification, reverse transcription, library construction, and sequencing were performed at Shanghai Majorbio Bio-pharm Biotechnology Co., Ltd. (Shanghai, China) according to the manufacturer’s instructions (Illumina, San Diego, CA, USA). The RNA-Seq transcriptome libraries were prepared using an Illumina TruSeqTM RNA Sample Preparation Kit (San Diego, CA, USA). RNA-Seq libraries were sequenced in a single lane on an Illumina Hiseq xten/NovaSeq 6000 sequencer (Illumina, San Diego, CA, USA) for 2 × 150 bp paired-end reads. The RNA-seq raw read data were processed using the software fastx_toolkit_0.0.14 (http://hannonlab.cshl.edu/fastx_toolkit/, accessed on 20 June 2021), SeqPrep (https://github.com/jstjohn/SeqPrep (accessed on 20 June 2021) and Sickle (https: //github.com/najoshi/sickle (accessed on 20 June 2021) to evaluate and discard sequences of low quality and those affected by adaptor contamination, with quality control shown in Supplementary Tables S2 and S3. The clean reads were mapped to the reference genome sequence of *Rosa chinensis*. The databases of NR, Swiss-Prot, Pfam, COG, GO, and KEGG were used for gene annotation.

## 5. Conclusions

This study reveals the effect of ^60^Co-γ irradiation on leaf morphology and physiological metabolism in *Rosa multiflora* ‘Libellula.’ Furthermore, we used RNA-seq to prove the differential expression of chlorophyll metabolism genes, ROS metabolism genes, and leaf development-related genes in irradiated leaves led to these changes. The VIGS technique also verified that *RcYABBY1* plays a vital role in development and morphogenesis of *R. multiflora* ‘Libellula’ leaves. Therefore, further study is needed to explore the molecular regulation mechanism of the interaction between genes and transcription factors in the regulation of leaf development after ^60^Co-γ irradiation mutagenesis. Our study on the mutation mechanism of mutants after irradiation treatment can provide theoretical support and effective guidance for *Rosa* irradiation breeding work.

## Figures and Tables

**Table 1:** Mutagenic effect of irradiation on leaf morphology.

Group Number	Variation Rate	Incised Leaves	Reduced Number of Compound Leaves	Rolling Leaf	Big Leaf	Biacuminate Leaf	Asymmetric Leaf
control	0	0	0	0	0	0	0
1-1	84.89 ± 5.18 de	17.55 ± 0.48 b	18.57 ± 0.94 d	22.92 ± 2.78 c	9.96 ± 0.46 b	5.89 ± 0.59 b	10.00 ± 1.11 cd
1-2	76.36 ± 1.18 e	26.45 ± 0.47 a	13.62 ± 0.98 e	12.80 ± 2.08 d	8.94 ± 1.11 bc	3.89 ± 0.48 c	10.66 ± 0.53 c
1-3	94.26 ± 4.43 d	3.02 ± 0.72 d	20.71 ± 0.86 c	48.70 ± 2.96 b	14.75 ± 1.52 ab	1.13 ± 0.21 de	5.95 ± 0.40 d
2-1	118.08 ± 4.46 b	15.32 ± 0.57 cd	32.49 ± 1.27 ab	48.35 ± 4.06 bc	5.81 ± 2.50 c	3.79 ± 1.01 cd	12.32 ± 0.77 b
2-2	106.36 ± 1.63 c	16.24 ± 0.39 c	33.53 ± 1.48 a	11.21 ± 2.55 de	16.41 ± 1.58 a	8.14 ± 1.01 a	20.83 ± 1.43 a
2-3	141.67 ± 2.71 a	17.11 ± 1.16 bc	27.28 ± 1.98 b	81.06 ± 2.09 a	4.53 ± 0.44 cd	1.87 ± 0.50 d	9.83 ± 1.30 cd

Note: Multiple changes were observed for some leaves. Data are presented as the mean ± standard. deviation (triplicate). Lowercase letters indicate significant differences between groups (*p* < 0.05).

## Data Availability

The data presented in this study are available upon request from the corresponding author.

## Supplementary Materials

The following supporting information can be downloaded at: https://www.mdpi.com/article/10.3390/plants11111438/s1, Figure S1: Irradiation treatment of *Rosa multiflora* ‘Libellula.’; Figure S2: Plant leaf phenotypes when counting leaf variation rates; Figure S3: PCA analysis of control and 2-3; Figure S4: qPCR analysis of RcKNOX1 in 2-1, 2-2 and 2-3; Table S1: Primers used in this study; Table S2: Quality control of the transcriptome analysis; Table S3: Mapping summary of the sequencing data for each sample; Table S4: Description of differently expressing heat-related genes shown in the heat map; Table S5: differentially expressing transcription factors in *Rosa multiflora* ‘Libellula.’ before and after Irradiation.;
