# Supplementary material for: Mutagenic Effect of 60Co γ-Irradiation on Rosa multiflora ‘Libellula’ and the Mechanism Underlying the Associated Leaf Changes
Source: Plants (Basel). 2022 May 28;11(11):1438. doi: 10.3390/plants11111438 (PMC9182980; doi:10.3390/plants11111438)
Supplement: Supplementary file 1 [file plants-11-01438-s001.zip › plants-1716381-supplementary.pdf]

Supplementary File

Table.S1 Primers used in this study.

Primer	Sequence
VIGS for *Rosa multiflora 'Libellula'* .	
**RcYABBY1-TRV2-1-F**	TGAGTAAGGTTACCGAATTCTGTGATACTGTTCTCGCGGTAAGC
**RcYABBY1-TRV2-1-R**	GACATGCCCGGGCCTCGAGGTTAACAACAGGTGGAGGCTTAGG
**RcYABBY1-TRV2-2-F**	TGAGTAAGGTTACCGAATTCTCACTCAGAATCTCCCGCAGGAGG
**RcYABBY1-TRV2-2-R**	GACATGCCCGGGCCTCGAGGCCAAAGTGGATGTGAGGGAAGTG
**qPCR**	
**qRcYABBY1-F**	GTTCTCGCGGTAAGCGTTCC
**qRcYABBY1-R**	TCCTCCTGCGGGAGATTCTG
**qRcARF18-F**	TCTGGTGGATGTGCCCAGTC
**qRcARF18-R**	CTCATCGGTTTCTCGTTCTG
**qRcARF9-F**	TGGAAGTCCCTCGCCATAAG
**qRcARF9-R**	AGTAGCTGAACGTGCATGAC
**qRcWOX8-F**	AGTCGAGCAACTCCGGAAAC
**qRcWOX8-R**	GCTGCCTTGCAGTTACCTTG
**qRcKNOX1-F**	TTTCAGACCGCTCCGAATTG
**qRcKNOX1-R**	TGGTTGGAGGCCTGAGAAGC

Table. S2 Quality control of the transcriptome analysis.

Sample	Clean reads	Clean bases	Error rate(%)	Q20(%)	Q30(%)	GC content(%)
2-3-1	54599482	8119232797	0.0249	98	94.26	46.59
2-3-2	51611548	7631256403	0.0251	97.95	94.15	46.19
2-3-3	49058954	7285603907	0.0252	97.89	94.01	46.33
CONTROL1	52821844	7827108418	0.0249	98.01	94.31	46.53
CONTROL2	44295440	6592723324	0.0253	97.81	93.89	45.99
CONTROL3	51431848	7619451321	0.0252	97.9	94.04	46.11

Table. S3 Mapping summary of the sequencing data for each sample

Sample	Total reads	Total mapped	Multiple mapped	Uniquely mapped
2-3-1	54599482	46384120(84.95%)	1559828(2.86%)	44824292(82.1%)
2-3-2	51611548	43702114(84.68%)	1664192(3.22%)	42037922(81.45%)
2-3-3	49058954	41672222(84.94%)	1569834(3.2%)	40102388(81.74%)
CONTROL1	52821844	44331028(83.93%)	1649705(3.12%)	42681323(80.8%)
CONTROL2	44295440	36913740(83.34%)	1377761(3.11%)	35535979(80.22%)
CONTROL3	51431848	42755788(83.13%)	1792291(3.48%)	40963497(79.65%)

Table. S4 Description of differently expressing heat-related genes shown in the heat map.

Abbreviation	Description
POD44-like	peroxidase 44-like
POD27-like	peroxidase 27-like
POD21	peroxidase 21
GST-like-1	glutathione S-transferase-like
POD64	peroxidase 64
APX3-like	L-ascorbate peroxidase 3-like, transcript variant X1
POD42	peroxidase 42
POD17-like	peroxidase 17-like
GST2	probable glutathione S-transferase
POD31-like	peroxidase 31-like
POD4-like	peroxidase 4-like
APX2	L-ascorbate peroxidase 2, cytosolic, transcript variant X3
GST3	probable glutathione S-transferase
POD7-like	peroxidase 7-like
GST-U10-like	glutathione S-transferase U10-like
POD73	peroxidase 73
GST-U17-like	glutathione S-transferase U17-like
SOD1	superoxide dismutase [Cu-Zn]
GST1	probable glutathione S-transferase
POD9-like	peroxidase 9-like
GST-like-2	glutathione S-transferase zeta class-like
NOL	chlorophyll(ide) b reductase NOL, chloroplastic
SAG12-like-2	senescence-specific cysteine protease SAG12-like
PPH	pheophytinase, chloroplastic, transcript variant X2
SGR-like	protein STAY-GREEN LIKE, chloroplastic
NYC1	probable chlorophyll(ide) b reductase NYC1, chloroplastic
PAO	pheophorbide a oxygenase, chloroplastic
CS1-like	chlorophyllase-1-like
SAG12-liek-1	senescence-specific cysteine protease SAG12-like
HEMG-like	protoporphyrinogen oxidase, mitochondrial-like, transcript variant X1
CHLD	magnesium-chelatase subunit ChlD, chloroplastic, transcript variant X1
HEMG1	protoporphyrinogen oxidase 1, chloroplastic

HEMA1	glutamyl-tRNA reductase-binding protein, chloroplastic
CHLG	chlorophyll synthase, chloroplastic
HEMA2-like	glutamyl-tRNA reductase 2, chloroplastic-like
CHLI	magnesium-chelatase subunit ChlI, chloroplastic
CHLH	magnesium-chelatase subunit ChlH, chloroplastic
CAO	chlorophyllide a oxygenase, chloroplastic, transcript variant X1
GSA1	glutamate-1-semialdehyde 2,1-aminomutase 2, chloroplastic-like
REV	homeobox-leucine zipper protein REVOLUTA
ARF18-like	auxin response factor 18-like
ARF9	auxin response factor 9
YABBY1-X1	axial regulator YABBY 1, transcript variant X1
YABBY1-X2	axial regulator YABBY 1, transcript variant X2
YABBY4	axial regulator YABBY 4
PIN-LIKES-7-X4	protein PIN-LIKES 7, transcript variant X4
PIN-LIKES-7-X2	protein PIN-LIKES 7, transcript variant X2
PIN-LIKES-7-X1	protein PIN-LIKES 7, transcript variant X1
PIN-LIKES-7-X3	protein PIN-LIKES 7, transcript variant X3
KNOX1-like-6	homeobox protein knotted-1-like 6
TCP7-like	transcription factor TCP7-like
PIN-LIKES-3-like	protein PIN-LIKES 3-like
TCP15	transcription factor TCP15
KNOX1-like-1-X2	homeobox protein knotted-1-like 1, transcript variant X2
ARF2-X1	auxin response factor 2, transcript variant X1
KNOX1-like-3-X1	homeobox protein knotted-1-like 3, transcript variant X1
KNOX1-like-3-X2	homeobox protein knotted-1-like 3, transcript variant X2
KNOX1-like-3-X3	homeobox protein knotted-1-like 3, transcript variant X3
TCP9-like	transcription factor TCP9-like
TCP4-like	transcription factor TCP4-like
TCP10-like	transcription factor TCP10-like
WOX8-like	WUSCHEL-related homeobox 8-like
YABBY5	axial regulator YABBY 5
KAN1-like	transcription repressor KAN1-like
WOX4	WUSCHEL-related homeobox 4
ARF6	auxin response factor 6
KAN4	probable transcription factor KAN4
KAN1-X1	transcription repressor KAN1, transcript variant X1
ARF19-like	auxin response factor 19-like
KAN2-X1	probable transcription factor KAN2, transcript variant X1
YABBY1-like	axial regulator YABBY 1-like
TCP9-like	transcription factor TCP9-like
ARF5-X1	auxin response factor 5, transcript variant X1

Table. S5 differentially expressing transcription factors in *Rosa multiflora* 'Libellula.' before and after Irradiation.

Gene_id	Log_2FC(2-3-1_leaf/control _leaf)	Pvalue	Regulate	NR description
gene16656	-1.528495628	0.000662024	down	XP_024186574.1(peroxidase 44-like [Rosa chinensis])
gene33076	-5.333884343	0.00018568	down	XP_024161518.1(peroxidase 27-like [Rosa chinensis])
gene14505	-2.748320462	0.003647732	down	XP_024189371.1(peroxidase 21 [Rosa chinensis])
gene32896	-1.708094906	0.005502435	down	XP_024166768.1(glutathione S-transferase-like [Rosa chinensis])
gene6849	-6.046292455	6.65783E-05	down	XP_024181444.1(peroxidase 64 [Rosa chinensis])
gene6883	-5.356215633	0.001230364	down	XP_024177575.1(L-ascorbate peroxidase 3-like [Rosa chinensis])
gene22160	-2.293398214	0.000403815	down	XP_024160380.1(peroxidase 42 [Rosa chinensis])
gene8004	-1.755000338	4.20109E-05	down	XP_024184937.1(peroxidase 17-like [Rosa chinensis])
gene32264	-1.564762578	0.000372258	down	XP_024163334.1(probable glutathione S-transferase [Rosa chinensis])
gene12246	-5.043066147	0.000121863	down	XP_024178048.1(LOW QUALITY PROTEIN: peroxidase 31-like [Rosa chinensis])
gene4623	-2.010046838	0.000172505	down	XP_024176145.1(peroxidase 4-like [Rosa chinensis])
gene32264	-1.729476716	0.004344474	down	XP_024163334.1(probable glutathione S-transferase [Rosa chinensis])
gene23682	-1.699709289	6.88855E-05	down	XP_024200026.1(peroxidase 7-like [Rosa chinensis])
gene21275	-2.067306535	0.005023484	down	XP_024196875.1(glutathione S-transferase U10-like [Rosa chinensis])
gene7589	-1.020652779	0.00335008	down	XP_024183424.1(peroxidase 73 [Rosa chinensis])
gene21708	-1.62835802	2.21653E-05	down	XP_024194287.1(glutathione S-transferase U17-like [Rosa chinensis])
gene13202	-2.005306517	0.001840375	down	XP_024186633.1(superoxide dismutase [Cu-Zn] [Rosa chinensis])
gene32264	-1.687570592	3.41102E-05	down	XP_024163334.1(probable glutathione S-transferase [Rosa chinensis])
gene38747	-2.382945813	4.00343E-05	down	XP_024172076.1(peroxidase 9-like [Rosa chinensis])
gene37972	-1.455107678	0.000695293	down	XP_024171440.1(glutathione S-transferase zeta class-like [Rosa chinensis])
gene1432	6.832933173	1.54809E-07	up	XP_024177789.1(chlorophyll(ide) b reductase NOL, chloroplastic [Rosa chinensis])
gene24673	5.54280401	7.95599E-06	up	XP_024159232.1(senescence-specific cysteine protease SAG12-like [Rosa chinensis])
gene29498	2.033738875	1.45904E-06	up	XP_024167155.1(protein STAY-GREEN LIKE, chloroplastic [Rosa chinensis])
gene6109	2.446768305	0.003440049	up	XP_024183818.1(probable chlorophyll(ide) b reductase NYC1, chloroplastic [Rosa chinensis])
gene26586	2.609086928	2.01843E-06	up	XP_024156933.1(pheophorbide a oxygenase, chloroplastic [Rosa chinensis])
gene32622	1.728760675	0.000863304	up	XP_024162290.1(chlorophyllase-1-like [Rosa chinensis])
gene24673	4.352665245	0.001234561	up	XP_024159232.1(senescence-specific cysteine protease SAG12-like [Rosa chinensis])
gene12610	5.201968684	2.88536E-05	up	XP_024181665.1(protoporphyrinogen oxidase, mitochondrial-like isoform X1 [Rosa chinensis])
gene26149	-5.154126459	5.15967E-06	down	XP_024158284.1(magnesium-chelatase subunit ChlD, chloroplastic isoform X1 [Rosa chinensis])
gene15819	-5.339303203	4.73707E-05	down	XP_024189297.1(protoporphyrinogen oxidase 1, chloroplastic [Rosa chinensis])
gene10698	-1.217763345	0.000618138	down	XP_024178168.1(glutamyl-tRNA reductase-binding protein, chloroplastic [Rosa chinensis])
gene13973	-1.010263478	0.001064966	down	XP_024188706.1(chlorophyll synthase, chloroplastic [Rosa chinensis])
gene30911	-1.990801888	0.002313869	down	XP_024165401.1(glutamyl-tRNA reductase 2, chloroplastic-like [Rosa chinensis])
gene22728	-2.119355568	0.000342817	down	XP_024157080.1(magnesium-chelatase subunit ChlI, chloroplastic [Rosa chinensis])
gene15557	-7.332430121	0.001246446	down	XP_024191253.1(magnesium-chelatase subunit ChlH, chloroplastic [Rosa chinensis])

gene33116	-1.332220595	6.58188E-05	down	XP_024166950.1(chlorophyllide a oxygenase, chloroplastic isoform X1 [Rosa chinensis])
gene35632	-1.020652779	0.00335008	down	XP_024171325.1(glutamate-1-semialdehyde 2,1-aminomutase 2, chloroplastic-like [Rosa chinensis])
gene26765	9.185511444	9.41323E-05	up	XP_024158223.1(homeobox-leucine zipper protein REVOLUTA [Rosa chinensis])
gene3690	-1.739482221	0.001122121	down	XP_024176234.1(auxin response factor 18-like [Rosa chinensis])
gene35359	-5.056479032	1.5434E-07	down	XP_024172879.1(auxin response factor 9 [Rosa chinensis])
gene2857	-7.145543988	0.002851183	down	XP_024183329.1(axial regulator YABBY 1 isoform X1 [Rosa chinensis])
gene21439	-4.497172244	4.73707E-05	down	XP_024196491.1(axial regulator YABBY 4 [Rosa chinensis])
gene6706	-2.507668546	8.08208E-05	down	XP_024180004.1(protein PIN-LIKES 7 isoform X1 [Rosa chinensis])
gene20749	-1.949280809	1.07067E-05	down	XP_024192416.1(homeobox protein knotted-1-like 6 [Rosa chinensis])
gene17899	-3.870222367	0.002038181	down	XP_024195153.1(transcription factor TCP7-like [Rosa chinensis])
gene37873	-1.68863356	0.000121852	down	XP_024174537.1(protein PIN-LIKES 3-like [Rosa chinensis])
gene21287	-1.33906977	0.005806803	down	XP_024194958.1(transcription factor TCP15 [Rosa chinensis])
gene34700	-1.589846749	0.000118662	down	XP_024168675.1(homeobox protein knotted-1-like 1 [Rosa chinensis])
gene35587	-7.97152299745	1.05969E-07	down	XP_024168835.1(auxin response factor 2 [Rosa chinensis])
gene33226	-2.054347264	3.1655E-05	down	XP_024162560.1(homeobox protein knotted-1-like 3 isoform X1 [Rosa chinensis])
gene3065	-1.629739659	0.00152087	down	XP_024173524.1(transcription factor TCP9-like [Rosa chinensis])
gene14213	-2.314834839	6.10171E-05	down	XP_024190152.1(transcription factor TCP4-like [Rosa chinensis])
gene36691	-1.523420273	0.000183523	down	XP_024171607.1(transcription factor TCP10-like [Rosa chinensis])
gene37751	-3.188478469	0.000459391	down	XP_024173293.1(WUSCHEL-related homeobox 8-like [Rosa chinensis])
gene5941	-1.835558935	0.000310247	down	XP_024179882.1(axial regulator YABBY 5 [Rosa chinensis])
gene21747	-2.318533916	2.12731E-05	down	XP_024195163.1(transcription repressor KAN1-like [Rosa chinensis])
gene34188	-1.728720241	0.005045593	down	XP_024166844.1(WUSCHEL-related homeobox 4 [Rosa chinensis])
gene23138	-1.650879371	0.002440906	down	XP_024199387.1(auxin response factor 6 [Rosa chinensis])
gene33478	-3.156087361	2.49237E-06	down	XP_024166549.1(probable transcription factor KAN4 [Rosa chinensis])
gene34682	-1.002150038	0.003924055	down	XP_024169806.1(transcription repressor KAN1 [Rosa chinensis])
gene17817	-1.697060373	7.67235E-05	down	XP_024193102.1(auxin response factor 19-like [Rosa chinensis])
gene37443	-4.599806805	0.002123738	down	XP_024172898.1(probable transcription factor KAN2 isoform X1 [Rosa chinensis])
gene36697	-1.550182777	0.000881593	down	XP_024173131.1(axial regulator YABBY 1-like [Rosa chinensis])
gene3065	-1.223767033	0.0017297	down	XP_024173524.1(transcription factor TCP9-like [Rosa chinensis])
gene33695	-4.18724116	0.003184845	down	XP_024166617.1(auxin response factor 5 isoform X1 [Rosa chinensis])

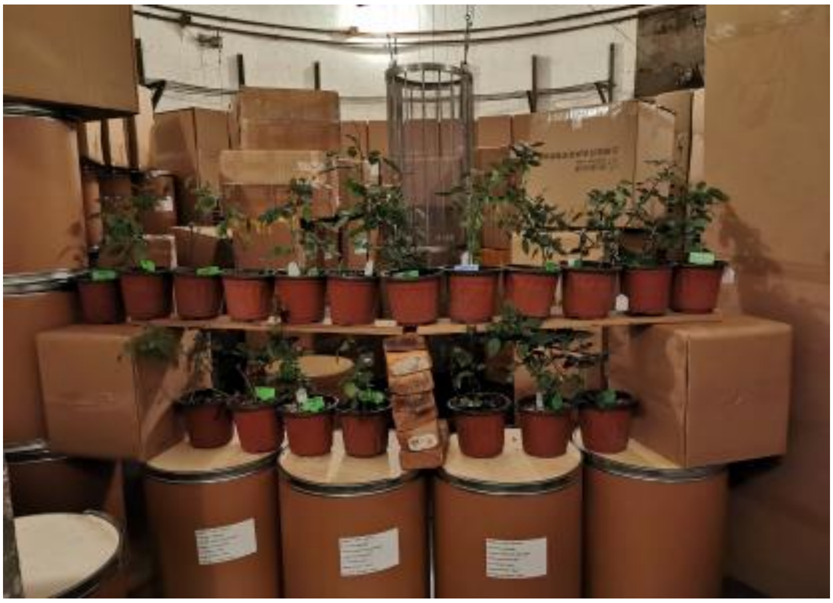

Figure S1 Irradiation treatment of *Rosa multiflora* 'Libellula.'

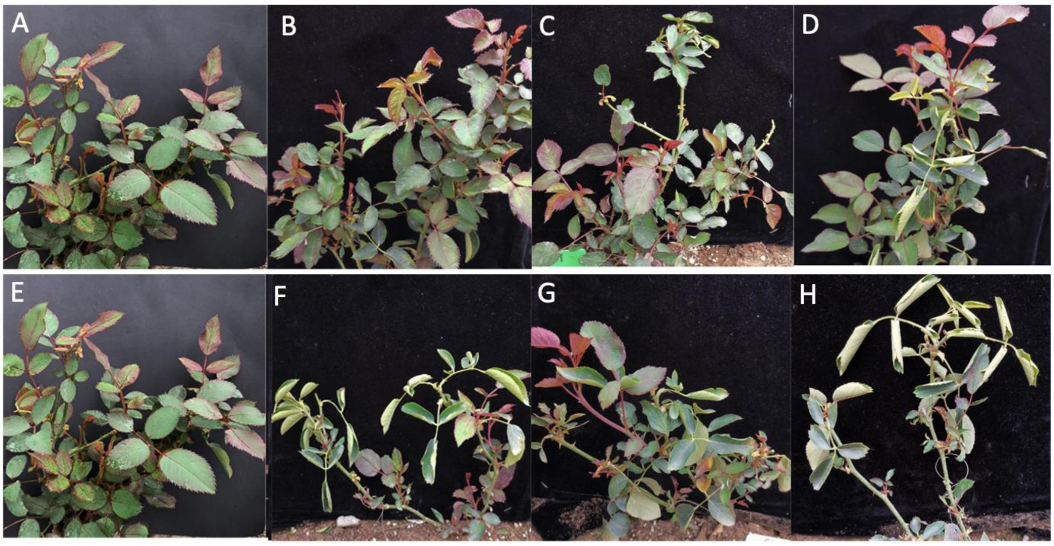

Figure S2 Plant leaf phenotypes when counting leaf variation rates. （A）control （B）1-1 （C）1-2 （D）1-3 （E）control（F）2-1 （G）2-2（H）2-3

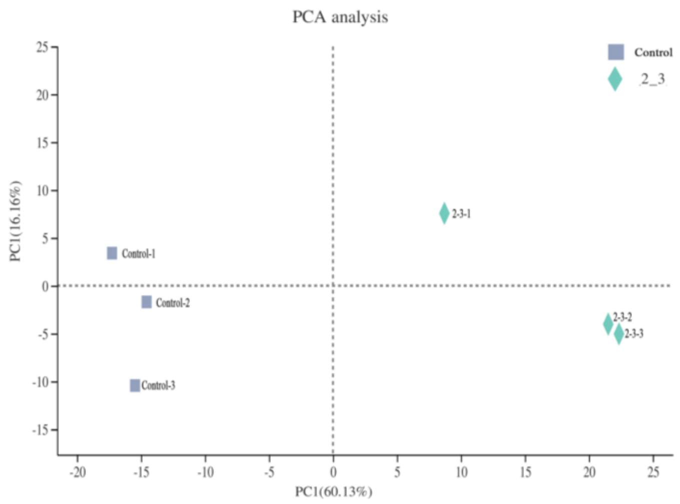

Figure S3 PCA analysis of control and 2-3.

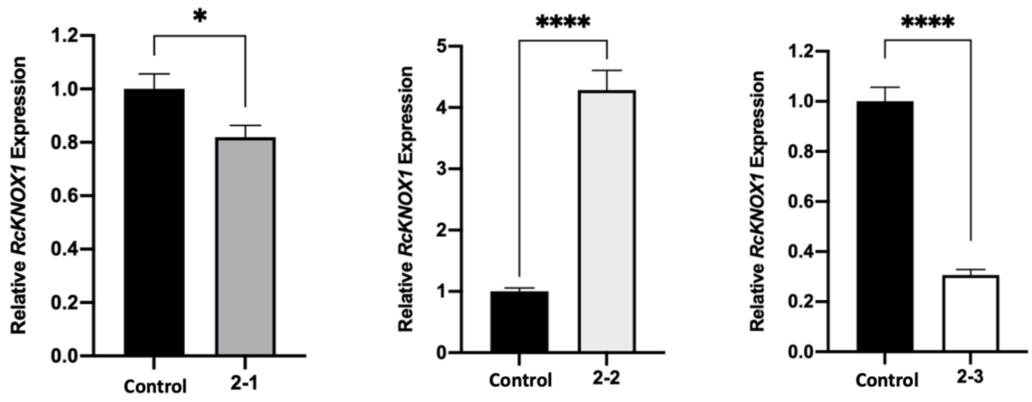

Figure S4 qPCR analysis of *RcKNOX1* in 2-1,2-2 and2-3. Asterisks indicate significant differences among treatments (* $p < 0.05$; **** $p < 0.0001$). The vertical bars indicate the standard deviations of the means of three tests.
